# Evaluation of Extended Storage of Swine Complete Feed for Inactivation of Viral Contamination and Effect on Nutritional, Microbiological, and Toxicological Profiles

**DOI:** 10.3390/ani14030393

**Published:** 2024-01-25

**Authors:** Jordan T. Gebhardt, Scott A. Dee, Erin Little, Brittney N. Scales, Doug R. Kern

**Affiliations:** 1 Department of Diagnostic Medicine/Pathobiology, College of Veterinary Medicine, Kansas State University, Manhattan, KS 66506, USA; 2 Pipestone Applied Research, Pipestone Veterinary Services, Pipestone, MN 56164, USA; scottdee7255@gmail.com (S.A.D.); erin.little@pipestone.com (E.L.); 3 eGenesis, Cambridge, MA 02140, USA; brittney.scales@egenesisbio.com (B.N.S.); doug@kernmail.org (D.R.K.)

**Keywords:** feed, PEDV, PRRSV, SVA, quality, swine, virus

## Abstract

**Simple Summary:**

The extended storage of feed ingredients has been suggested as a method to mitigate the risk of viral transmission through contaminated ingredients. To validate the approach of extended storage of complete swine feed for inactivation of swine viruses, an experiment was conducted to evaluate the ability of extended storage to inactivate the porcine reproductive and respiratory syndrome virus (PRRSV), porcine epidemic diarrhea virus (PEDV), and Senecavirus A (SVA) when stored for 58 d at 23.9 °C using a self-contained intermodal shipping container. Minimal detrimental impacts were observed when storing feed for 58 d on feed quality measurements including mycotoxin detection, identification and quantification of bacteria, and stability of vitamins and phytase. No infectivity was observed for PRRSV, PEDV, or SVA following 58 d storage at 23.9 °C with the exception of a documented biocontainment breach for PRRSV. The results indicate that the extended storage of complete swine feed can be a method to reduce risks associated with pathogen transmission through feed while having minimal effects on measures of nutritional quality.

**Abstract:**

The extended storage of feed ingredients has been suggested as a method to mitigate the risk of pathogen transmission through contaminated ingredients. To validate the approach of extended storage of complete swine feed for the inactivation of swine viruses, an experiment was conducted wherein swine feed was inoculated with 10 mL of 1 × 10^5^ TCID_50_/mL of porcine reproductive and respiratory syndrome virus (PRRSV), porcine epidemic diarrhea virus (PEDV), and Senecavirus A (SVA) and stored for 58 d at 23.9 °C. Measures of feed quality were also evaluated at the initiation and conclusion of the storage period including screening for mycotoxins, characterization of select microbiological measures, and stability of phytase and dietary vitamins. Storing feed for 58 d under either ambient or anaerobic and temperature-controlled storage conditions did not result in substantial concerns related to microbiological profiles. Upon exposure to the feed following 58 d of storage in a swine bioassay, previously confirmed naïve pigs showed no signs of PEDV or SVA replication as detected by the PCR screening of oral fluids and serum antibody screening. Infection with SVA was documented in the positive control room through diagnostic testing through the State of Minnesota. For PRRSV, the positive control room demonstrated infection. For rooms consuming inoculated feed stored for 58 d, there was no evidence of PRRSV infection with the exception of unintentional aerosol transmission via a documented biocontainment breach. In summary, storing feed for 58 d at anaerobic and temperature-controlled environmental conditions of 23.9 °C validates that the extended storage of complete swine feed can be a method to reduce risks associated with pathogen transmission through feed while having minimal effects on measures of nutritional quality.

## 1. Introduction

Contamination of feed ingredients or complete feed has been shown to be a potential route of pathogen transmission [1,2]. To control this potential route of disease transmission, efforts can focus on the prevention of the contamination step from occurring or implementing strategies to reduce the survival of the bacteria or virus in the material, and thus reduce the likelihood of causing clinical disease when presented to a naïve animal. Prevention strategies focus on biosecurity practices such as ingredient sourcing, controlling points of entry within the feed manufacturing system such as the ingredient-receiving pit, and implementing measures to reduce the likelihood of personnel or other fomites unintentionally introducing the virus into an ingredient or complete feed.

In the scope of feed biosecurity, after prevention practices have been implemented, a common follow-up step is the incorporation of active mitigation techniques to reduce pathogen survival and/or ability to cause infection when exposed to susceptible animals. Multiple practices have been shown to reduce the survival of pathogens including bacteria and viruses in ingredients and/or complete feed including thermal processing [3], chemical feed additives [4,5,6,7,8,9,10], and irradiation [8]. While these processes can all reduce viral survival, their use in different practical settings is largely dependent on a number of factors including cost, availability, level of risk aversion desired by the customer, and impact on nutritional components such as vitamins and enzymes. Historically, irradiation has been shown to be a highly effective method to reduce the contamination of feed ingredients or complete feed [8]. Given the high level of efficacy for feed sanitation, irradiation has been commonly used in biomedical research settings to ensure that the feed provided to animals is free of pathogenic agents. However, potential negative consequences of irradiation include significant financial cost, degradation in nutrients including vitamins, and limited throughput based on current infrastructure. Furthermore, research has explored the extended storage of inoculated feed ingredients to reduce viral detection and infectivity. These studies have demonstrated the viral inactivation characteristics of multiple viruses in a variety of feed ingredients and complete feed under different conditions [11,12].

Research has extended beyond the laboratory and has used real-world conditions to evaluate the stability of SVA in soybean meal using 1 metric ton volumes using a temperature of 23.9 °C and a storage of 30 d [13]. However, since feed has the potential for contamination during the milling and delivery phases of the supply chain, additional validation is needed for the extended storage of complete swine feed to inactivate viruses on a practical scale. Thus, exploring potential strategies to achieve high levels of efficacy using alternative methods to irradiation would be useful for a number of industries, including biomedical research using pigs as a research model, given their higher feed intake relative to smaller research animals such as rodents. Previous research has established that a 30 d holding period was effective to inactivate SVA when stored at 23.9 °C [13], and within this experiment it was decided to evaluate viral inactivation and feed quality with a storage period approximately twice as long as the previously established holding guidelines. Our hypothesis is that the extended storage of complete swine feed will result in viral inactivation and will maintain satisfactory levels of nutrient stability. Thus, the objective of this experiment was to validate a 58 d anaerobic and temperature-controlled extended storage protocol to determine the effect on the nutritional quality of complete feed and for inactivation of three swine viruses (PEDV, PRRSV, SVA) when inoculated into complete swine feed.

## 2. Materials and Methods

### 2.1. General

To store swine feed during the extended storage period, custom center-flow solid polyethylene containers (dimensions: 1.4 × 1.0 × 0.8 m; RPP Containers, Cincinnati, OH, USA) were used, each having a capacity of approximately 450 kg per container. A climate-controlled intermodal shipping container was used to create the specified environmental storage conditions (A&M Cold Storage and Trailer Leasing, Suwanee, GA, USA; 12.2 m long × 2.3 m wide × 2.6 m tall). The intermodal shipping container was fitted with a self-contained heating and refrigeration unit that operated off of a 480 volt, 3-phase power supply. The dimensions of the feed storage containers were designed so that the intermodal shipping container could store 28 feed totes (14 per layer; two layers high). 

**Figure 1:**
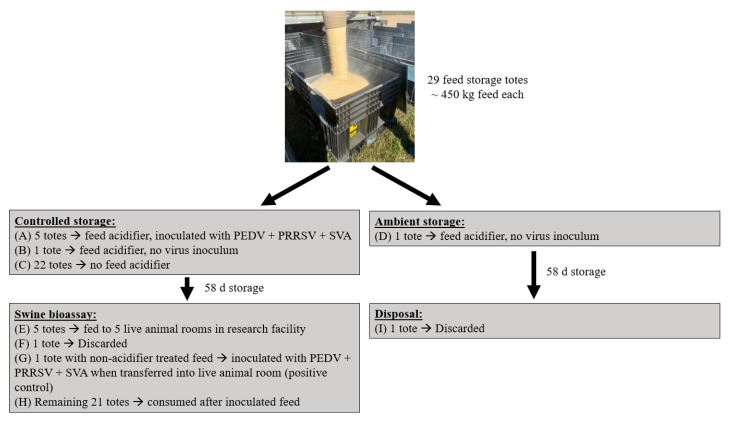
Experimental design.

**Figure 2:**
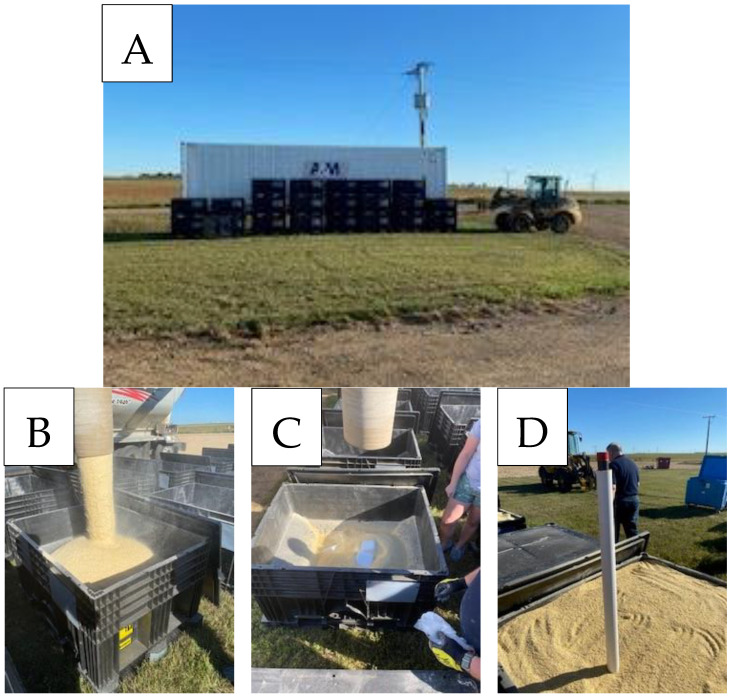
Equipment and processes. (**A**) Intermodal shipping container along with feed storage containers; (**B**) filling of feed storage containers; (**C**) adding dry ice to feed storage container after bottom surface has been covered with base layer of feed; and (**D**) viral inoculation technique once feed storage totes have been filled.

This study used a commercial formulation of swine finishing diet (Table 1). The dietary formulation was a standard corn–soybean meal-dried distillers grains with solubles-based diet. A subset of the feed contained a commercially available blend of formic and propionic acid (0.3% inclusion; pHorce, Anpario, Nottinghamshire, UK). The dietary acidifier was included in virus-inoculated feed totes and feed totes used to assess microbiological, toxicological, and nutritional profiles. The remainder of the feed which was used to feed pigs within the bioassay following consumption of the virus-inoculated feed did not contain feed additives and corn was added at the expense of the dietary acidifier. Diets were manufactured at a commercial feed mill (Chandler Feeds, Woodstock, MN, USA) and transported to the biosafety level 2 (BSL-2) Pipestone Research facility. Upon arrival at the facility, a small amount of feed was transferred into the bottom of each container; then, a 2.26 kg block of dry ice was added to the storage container, and the container was then filled to approximately 450 kg of feed per container. A total of 29 feed storage containers were used, each containing approximately 450 kg of swine feed (Figure 1). Methods used in the experiment are shown in Figure 2.

### 2.2. Inoculum Preparation and Tote Inoculation

Following transfer of feed into feed storage containers, 5 containers were inoculated with viral inoculum prior to being sealed. Viral inoculum consisted of PRRSV-144 L1C variant, PEDV, and SVA and was confirmed to be viable through the propagation and titration procedures at South Dakota State University. Selection of these viruses was based on the recent emergence of the PRRSV-144 L1C variant, history of PEDV in feed biosecurity research, and stability of SVA in feed ingredients and complete feed. Inoculation procedures were identical to those previously described [13], with the addition of PEDV in the current experiment. Briefly, each of the 3 viruses was prepared into a 10 mL ice cube containing a mixture of each virus with a total dose of 1 × 10^5^ TCID_50_ per virus. Each virus was diluted in 30 mL of minimum essential medium (MEM, Sigma-Aldrich, St. Louis, MO, USA). Ice cubes were prepared by freezing 10 mL aliquots of the mixture in 50 mL conical centrifuge tubes (Corning Inc., Corning, NY, USA) at −80 °C. 

To inoculate totes, a solid metal rod (1.2 m length × 4.45 cm diameter) was inserted into the lumen of a PVC pipe (1.2 m length × 5.08 cm diameter). The combined rod and pipe “inoculation instrument” was manually forced into the middle of each tote. Once the instrument was in the proper place, the internal metal rod was removed, providing a clear path for the ice cube to travel into the interior of the tote, unimpeded by the presence of meal. Following the removal of the rod, the 10 mL ice cube was dropped into the PVC tube, the PVC tube was removed, resulting in the cube being buried by the meal and creation of the “hot spot” centralized in the tote.

### 2.3. Storage Procedure

The containers had solid plastic lids attached and were stored outside overnight to allow excess CO_2_ to sublimate for human health considerations when transferring into a confined airspace of the intermodal shipping container. Following overnight storage in open air, feed storage containers were transferred to their appropriate storage location. A total of 28 totes were transferred into the intermodal shipping container to fill to full capacity. Five totes contained feed treated with the commercial feed acidifier and treated with the viral inoculum, one tote was transferred and contained feed treated with the commercial feed acidifier and no inoculation with virus to serve as a source of feed to be used to assess stability of nutrients including vitamins and enzyme stability. This tote was used so no known viral-inoculated feed would be sent to laboratories for nutritional analysis. Additionally, 22 totes were transferred into the intermodal shipping container which contained feed not treated with the commercial feed acidifier and this feed was used to maintain pigs during the bioassay following consumption of the viral-inoculated feed. Following placement of the feed in the intermodal shipping container, approximately 45 kg of dry ice was placed between the feed storage containers, the door was sealed, and a vent located at the top of the intermodal shipping container was kept open for 12 additional hours to allow any pressure to escape as the dry ice continued to undergo sublimation and the denser CO_2_ displaced air out of the discharge vent. After this period of venting excess pressure, the container was fully sealed, and the integrated refrigeration unit was set to a target temperature of 23.9 °C based on previous research [13], which would be automatically maintained over the storage period.

In addition to the feed storage totes under anaerobic and temperature-controlled storage conditions in the intermodal shipping container, one tote was placed in an uninsulated machinery storage shed to characterize the nutritional stability in uncontrolled environmental storage conditions. This study was conducted in southwest Minnesota from approximately September through October 2022, which resulted in significant fluctuation in ambient conditions. 

### 2.4. Environmental Monitoring during Storage

During the process of filling feed storage containers, temperature and relative humidity (% RH) data loggers (RC-51H waterproof USB temperature data logger, Elitech, San Jose, CA, USA) were placed to characterize storage conditions with data recorded every 10 min continuously throughout the storage period. A total of 11 data loggers were used, with six being placed within feed storage totes stored in controlled conditions and one within the feed storage tote stored in ambient conditions to characterize the conditions within the feed. In addition to monitoring conditions within feed, three data loggers were placed in the intermodal shipping container approximately 11.5 cm from the floor and distributed evenly within the intermodal shipping container to characterize air temperature and humidity over the storage period. And a final data logger was placed in the machinery storage shed to characterize the conditions for the tote stored in ambient environmental conditions. 

### 2.5. Feed Sampling and Quality Measurements

Feed samples were collected on d 0 from the feed storage tote which was stored at ambient conditions and from the feed storage tote to be stored in controlled environmental conditions. Feed samples were collected from totes containing feed treated with the commercial blend of formic and propionic acids. Feed was sampled using a feed probe to collect 10 samples per feed storage container and were mixed to create a composite sample for each container. This sample process was repeated following the 58 d storage period. Feed samples from d 0 and d 58 were analyzed at the North Dakota State University Veterinary Diagnostic Laboratory using liquid chromatography–tandem mass spectrometry (LC/MS/MS). Feed samples were also analyzed to characterize microbiological profiles by assessing for concentration of yeast and mold (FDA BAM Chapter 18), aerobic plate count (AOAC 966.23), *Salmonella* (AOAC-RI 121501), *Escherichia coli* O157:H7 (AOAC-RI 031002), *Staphylococcus aureus* (FDA BAM Chapter 12), and *Clostridium perfringens* (ISO 7937). Feed samples collected on d 0 as well as feed samples stored for 58 d at ambient, controlled, and refrigerated (4 °C) conditions were analyzed at the DSM Nutritional Products Technical Marketing Analytical Services Laboratory (Belvidere, NJ, USA) for vitamins A, B5, D3, E, and phytase activity.

### 2.6. Virus Infectivity Assessment

Following the 58 d storage period, assessment of viral infectivity was performed using a BSL-2 live animal research facility. This facility had six rooms, each with its own feed bin, allowing for an individual tote to be allocated to a specific bin and be fed to a specific group of pigs. These rooms were organized into separate airspaces using filtration of incoming and outgoing air, with separate shower in/out, designated clean and dirty areas with a clear line of separation, along with the use of separate clothing, footwear, disposable gloves, and equipment per room to prevent room-to-room cross-contamination.

At the conclusion of the 58 d storage period, five sets of totes were transferred into five separate feed bins. A sixth room was used as a positive control where a feed tote was transferred into the feed bin with feed that was identical in formulation with the exception of not containing the commercial feed additive blend containing formic and propionic acid. The same viral inoculum was then added directly to the feed auger at the bottom of the bin and transferred into the live animal room using the automated feeding system. 

Each room was filled with approximately 30 pigs originating from a PRRSV, SVA, and PEDV-negative farm. Following consumption of the test feed, the pigs were fed diets that did not contain the formic/propionic acid commercial feed additive for the remainder of the study. During the 28-d bioassay, all six rooms of pigs were assessed for evidence of SVA and PRRSV 144 L1C infection through the collection of oral fluids (one rope per pen, six ropes per room) at d 0 (arrival), 14, and 30 of each replicate. In addition, daily observations were conducted, looking for clinical signs of SVA infection (vesicles and lameness) and signs of PRRSV 144 L1C variant infection (pyrexia, hyperemia, dyspnea, and weight loss). Selected cases of mortality were necropsied, lymphoid tissues collected, and tested by PCR for the presence of SVA and PRRSV RNA. Serum samples were analyzed for the presence of PEDV (fluorescent focus neutralization assay, PEDV USA/Colorado/2013 used as indicator virus), PRRSV (X3 Elisa), and SVA (IFA) antibodies. Oral fluid samples were analyzed using RT-PCR for detection of viral RNA. All diagnostic testing was performed at the Iowa State University Veterinary Diagnostic Laboratory. 

### 2.7. Statistical Analysis

Data were reported using appropriate statistical tests when possible and using descriptive statistics when sufficient variability was not present to satisfy the assumptions of ANOVA. Visualization of the temperature and humidity data over the storage period was performed using the ggplot2 package in R using the ggplot function (version 4.2.1; 23 June 2022). For vitamin stability analysis, data were analyzed as a one-way ANOVA using the lmer function from the lme4 package in R with sample storage condition as a fixed effect. An individual feed sample was considered the experimental unit. Degrees of freedom were approximated using the Kenward–Roger approach and pairwise comparisons were performed using a Tukey multiple comparison adjustment. Results are considered significant at *p* ≤ 0.05 and marginally significant from *p* > 0.05 to *p* ≤ 0.10.

## 3. Results

### 3.1. Storage Conditions

**Figure 3:**
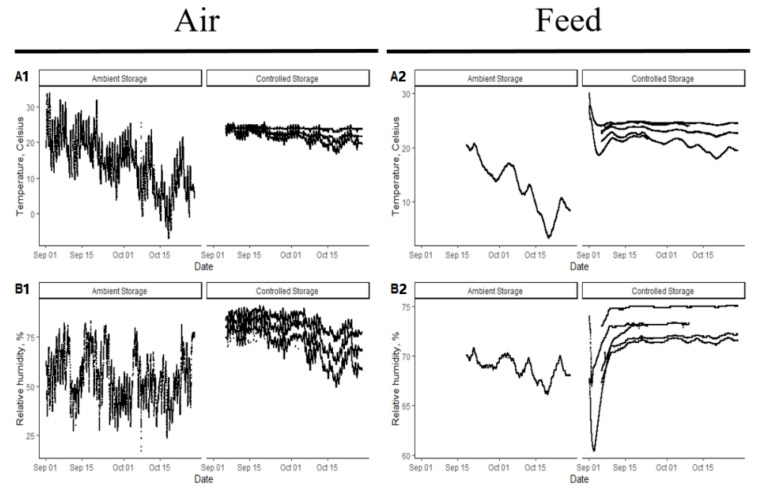
Temperature (**A1**) and relative humidity (**B1**) of air in ambient or controlled storage conditions. Temperature (**A2**) and relative humidity (**B2**) of feed stored in ambient or controlled storage conditions.

The environmental conditions for the ambient and controlled storage conditions are shown in Figure 3. Under controlled storage, the thermostat was set to 23.9 °C and the temperature remained relatively consistent over the duration of storage. There were differences observed based on the location of the data logger within the intermodal shipping container based on restricted airflow as the shipping container was completely full of feed storage totes, which likely limited air circulation. Relative humidity also differed based on the data logger location within the intermodal shipping container and there was a general reduction in relative humidity over time. Under ambient storage conditions, there was a high degree of variation in temperature and humidity within day as well as over the entire duration of the extended storage. Drastic differences in temperature and relative humidity were observed, as would be expected with no environmental control, and the temperature decreased over time, as would be expected based on geographic region and time of the year progressing through the fall months.

Temperature and relative humidity were also measured within the feed storage totes. Under controlled storage, there was variability in temperature and humidity between the feed storage totes evaluated, and this is likely due to the tote position within the intermodal shipping container. However, the feed remained at a more consistent temperature and relative humidity over time in the controlled storage conditions compared to the ambient storage conditions, as would be expected based on the environmental data. Over the course of the experiment, the temperature of the feed stored under ambient conditions decreased reflecting the environment, whereas the feed in the controlled storage remained consistent in terms of temperature and humidity.

### 3.2. Mycotoxin Analysis

To validate the safety of the feed following extended storage, a series of assays were conducted to look at mycotoxin concentrations as well as to evaluate the stability of key nutritional components including vitamins and the phytase enzyme. Samples collected at the beginning of the experiment and following 58 d of storage in either ambient or anaerobic and temperature-controlled conditions did not contain any detectable mycotoxins (Table 2). On d 0, there were detectable levels of yeast in one of two feed samples (120 cfu/g), mold in both feed samples (2700–2800 cfu/g), as well as aerobic plate counts ranging from 2000 to 5300 cfu/g. These levels are within the range of expected parameters for commercial swine feed based on the author’s experience with this diagnostic testing. Following the 58 d storage at ambient conditions, there was a slight increase in the detection of yeast (490 cfu/g) relative to d 0 samples, similar aerobic plate count (2200 cfu/g), and reduced mold concentration (320 cfu/g). Following 58 d of controlled storage, there was no detectable yeast (<10 cfu/g), and the concentration of mold (90 cfu/g) and aerobic plate count (260 cfu/g) were both lower than samples collected on d 0. Thus, it did not appear that storage of complete feed for 58 d under either ambient or controlled anaerobic and temperature-controlled storage conditions resulted in substantial concerns related to microbiological profiles.

### 3.3. Nutritional Analysis

For analysis of the concentration of the phytase enzyme, the analyzed content was 175% of the calculated claim of concentration on d 0 (Table 3). Following 58 d storage either at refrigerated temperatures, anaerobic and temperature-controlled storage in the intermodal shipping container, or under ambient conditions, there was a reduction (*p* < 0.05) in phytase activity compared to d 0. However, there were no differences between the three storage conditions (*p* > 0.05) for phytase activity when stored for 58 d. For vitamin A, the analyzed concentration on d 58 for feed samples stored in controlled or ambient conditions had greater (*p* < 0.05) vitamin A concentration compared to the d 0 baseline, and feed stored for 58 d under ambient conditions had greater (*p* < 0.05) vitamin A concentration compared to feed stored at 4 °C for 58 d. 

There is substantial variation associated with vitamin A analysis. This variation can be caused by variation due to sample collection, as well as laboratory analytical variation. Following 58 d storage at either controlled or ambient conditions, the concentration of vitamin A was still near the calculated claim for vitamin A concentration, indicating there was not a significant reduction in the concentration over time. For vitamin B5, there was a reduction in concentration following 58 d of storage at 4 °C, ambient, and controlled conditions compared to the d 0 baseline (*p* < 0.05), but concentrations were still near formulated claim levels following this period of storage. There was no evidence of changes (*p* > 0.05) in vitamin D3 or vitamin E concentration following 58 d of storage at any of the conditions evaluated.

### 3.4. Viral Stability during Storage

Following extended storage, inoculated feed samples were fed to pigs to determine whether infectious PRRSV, PEDV, or SVA was present. On d 0, there was no evidence of serum antibodies for any of the three viruses (Table 4). On d 27 of the bioassay, antibodies were detected for PRRSV in the positive control room (virus inoculated into feed immediately prior to feeding) as well as the adjacent room. There were no serum antibodies detected on d 27 for PEDV or SVA. Over the course of the experiment, there was no PEDV or SVA RNA detected in oral fluids on any sampling day (0, 6, 14, 20, and 27 dpi). For PRRSV, RNA was detected in three of six rooms (rooms 2, 3, and 5) on 0 dpi, indicating the presence of fragments of viral RNA within the feed and thus oral fluids of pigs sampled. However, in rooms 2 and 3, no subsequent oral fluid samples and the sample collected from room 5 on 6 dpi did not contain detectable PRRSV RNA, indicating no evidence of infection and confirming that the virus detected on 0 dpi was not infectious. For the positive control room, RNA was detected in oral fluids beginning on 6 dpi and all subsequent samples collected at 14, 20, and 27 dpi contained PRRSV RNA, indicating that the infectious virus was present in the inoculated feed sample. On 14 dpi, PRRSV RNA was detected in room 5, which was adjacent to the positive control room. This detection was attributed to a biosecurity breach within the facility and unintentional airflow was discovered between those two rooms. Thus, based on the lack of PRRSV RNA detection in room 5 on 6 dpi and subsequent detection beginning on 14 dpi, it appears that the infectious virus had spread from the positive control room into room 5 via this air leak.

No SVA RNA or antibodies to SVA were detected in any of the experimental rooms including the positive control room. However, clinical signs were present in the positive control room and through diagnostic testing through the State of Minnesota confirmed the presence of lesions consistent with SVA and detection of viral RNA in clinical samples from the positive control room, indicating that the infectious virus was present in the inoculated positive control feed.

## 4. Discussion

Contamination of feed ingredients before milling or complete feed during or post milling and delivery has been shown to be a potential route of pathogen transmission [1,2]. To control this potential route of disease transmission, efforts can focus on the prevention of the contamination step from occurring or implementing strategies to reduce the survival of the bacteria or virus in the material, and thus reduce the likelihood of causing clinical disease when presented to a naïve animal. Prevention of pathogen introduction and strategies that can be implemented have been previously described [14]. Strategies to reduce the survival of pathogens in feed ingredients or complete feed include thermal processing through strategies such as pelleting of complete diets [3], chemical feed additives [4,5,6,7,8,9,10], irradiation [8], and extended storage [13]. While these processes all can reduce viral survival, their use in different practical settings is largely dependent on several factors including cost, availability, and the level of risk aversion desired by the customer. 

Historically, the irradiation of feed ingredients and complete diets has been shown to reduce the concentration of bacteria within feed and improves growth performance [15,16]. Additionally, irradiation has been shown to inactivate swine viruses including PEDV [8,17]. Given the high degree of efficacy for feed sanitation, irradiation has been commonly used in biomedical research settings to ensure that feed provided to animals is free of pathogenic agents. However, potential negative consequences of irradiation include significant financial cost, degradation of nutrients including vitamins [18,19], and limited throughout based on current infrastructure. 

Furthermore, research has explored the extended storage of inoculated feed ingredients to reduce viral detection and infectivity. These studies have characterized the viral inactivation characteristics of multiple viruses in a variety of feed ingredients and complete feed under different conditions [11,12]. Research has extended beyond the laboratory and has used real-world conditions to evaluate the stability of SVA in soybean meal using 1 metric ton volumes [13]. However, additional validation is needed for extended storage of complete swine feed to inactivate viruses on a practical scale. In the current experiment, our goal was to build upon the previous literature and validate the extended storage of swine complete feed for the inactivation of swine viruses for potential use in settings such as biomedical research to provide alternative efficacious techniques to maintain feed safety using techniques other than irradiation.

As expected, storing complete swine feed under anaerobic and temperature-controlled environmental conditions reduced the variation in temperature and humidity compared to storage in ambient environmental conditions. It is hypothesized that maintaining a constant temperature and humidity would preserve feed quality compared to fluctuations in temperature and humidity, although little evidence is available currently. In the current experiment, there was no evidence of mycotoxin production over the 58 d storage period in either ambient or controlled environmental conditions. Furthermore, there was limited evidence of an increase in detrimental microbiological measures over the 58 d storage in either ambient or controlled storage conditions. The yeast concentration increased over the 58 d storage period for feed stored in ambient environmental conditions, but the measured concentration of 490 cfu/g is still within common values observed in commercial swine feed, and yeast is also commonly added as a feed additive in commercial swine diets [20]. The observed levels would not be expected to cause any detrimental health outcomes. 

Phytase is a common enzyme used in swine production to increase the utilization of plant-origin phosphorous in the form of phytase-bound P. Phytase stability is a critical measurement and its recovery decreases after extended storage [21] and following thermal processes such as pelleting [22,23]. It is common for the measured concentration of phytase to be well-above formulated levels as a margin of safety given these decay properties. In the current study, the analyzed concentration of phytase on d 0 was approximately 175% of the formulated value. There was a decay in the phytase activity following the 58 d storage period in all storage conditions, but the concentration of phytase was still approximately 100% of formulated claim following this period of extended storage. Thus, the results observed in the current study are consistent with the previous literature and illustrate that the phytase enzyme does lose activity following extended periods of storage which must be considered when implementing extended storage practices.

There is significant variation when measuring dietary vitamin concentration in finished feed attributed to both sampling and analytical variation. In the current experiment, there was evidence of decay over time for vitamin B5, but no evidence of reduced vitamin activity over time for vitamin A, D3, or E under any storage conditions. 

Previous research evaluated the stability of vitamins in vitamin premixes and observed that vitamin A is one of the most unstable vitamins in premixes, while vitamins D3, B5, and E were rather stable [24]. Similar results were observed by other researchers who observed that vitamin B5 was rather stable when stored in vitamin and vitamin-trace mineral premixes [25]. In the current experiment, vitamin B5 demonstrated a greater loss over the 58 d storage period compared to the other vitamins evaluated. Previous research also evaluated the stability of vitamin A in vitamin premixes and observed a high degree of stability over time up to 1 year of storage [26], which is similar to the results of the observed study albeit it a much shorter storage duration was investigated, and storage form was different (vitamin premix vs. complete swine feed). Data also exist indicating that vitamin form can have a significant impact on stability during storage. Particularly for vitamin A, there are multiple forms available including a cross-linked beadlet form and non-cross-linked forms. The cross-linked form has greater stability [27], and this was the form that was used in the current experiment. Although there are data evaluating vitamin stability over extended storage periods, much of the research has focused on the practical storage of vitamin and vitamin-trace mineral premixes and not the extended storage of complete feed.

From the perspective of measurements of feed quality, we did not observe significant detrimental effects of storing complete feed for 58 d at controlled or ambient conditions. It is important to consider, however, that the current experiment was aimed to validate practical conditions which included the use of a commercial feed additive containing a blend of formic and propionic acids as well as the creation of a semi-anaerobic environment using dry ice (solid CO_2_) when filling feed storage totes. Additional research is needed to fully understand the impact of feed storage on nutrient stability and microbiological profiles.

Following this period of extended anaerobic and temperature-controlled storage, an important goal was to validate inactivation of PEDV, PRRSV, and SVA. For PRRSV, evidence of infection was observed in the positive control room as expected, validating that including viral inoculum into feed immediately prior to feeding caused infection. There was a detection of viral RNA in oral fluids on 0 dpi, which indicates the presence of viral RNA in the feed but did not lead to infection, as documented by the lack of antibody production over time in those rooms as well as the lack of further detection of PRRSV RNA in oral fluids at later sampling points. There was detection of PRRSV infection in a room which was fed feed inoculated with all three viruses and stored for 58 d at 23.9 °C. Following closer investigation, a biosecurity gap was identified as a faulty seal between the positive control room and that test room which allowed unintentional airflow between the rooms. Due to the lack of infection documented on 6 dpi as measured using oral fluid samples, it appears that this biosecurity breach was the cause for the infection in that test room. This represents a limitation of this study. However, the four other test rooms (rooms 1–4) did not display any evidence of infection, giving a reasonable amount of confidence that the identified biosecurity breach was the cause of infection in test room 5 and not the extended storage of the feed. Nonetheless, this presents a limitation to the study and viral survival over the 58 d holding period cannot be completely ruled out.

A limitation of the current experiment was that there was no detection of viral RNA for PEDV in any of the test rooms along with no detectable levels of antibodies throughout the experiment. The viruses used in the current experiment were similar to those used in previous research [10]. In the current experiment, a total inoculum dosage of 1 × 10^5^ TCID_50_ per virus was inoculated into each feed tote and directly into the feed bin for the positive control room (approximately 0.1 TCID_50_ per gram of feed). In the previous work by Dee et al. [10], a total of 1 × 10^7^ TCID_50_ of PEDV, PRRSV, and SVA was inoculated into 1300 kg of complete feed (7.69 TCID^50^ per gram of feed for each virus). This difference in initial viral concentration may partially explain the lack of infectivity with PEDV in the current experiment, as the work by Schumacher et al. [28] which demonstrated that a concentration of 5.6 × 10^1^ TCID_50_ was required to cause infection under the conditions used. For SVA, however, clinical signs were present in the positive control room and through diagnostic testing through the State of Minnesota confirmed the presence of lesions consistent with SVA and the detection of viral RNA in clinical samples from the positive control room, indicating that the infectious virus was present in the inoculated positive control feed. For SVA, Buckley et al. [29] reported that approximately 6.3 × 10^2^ TCID_50_ was required to cause infection when the dose was provided via liquid inoculum, and Caserta et al. [30] reported that a dose of 1 × 10^5^ TCID_50_, 1 × 10^6^ TCID_50_, and 1 × 10^7^ TCID_50_ all caused infection through inoculated feed. The dose used in the current experiment was 1 × 10^5^ TCID_50_, which was sufficient to cause clinical infection, but did not stimulate the production of antibodies or result in shedding via oral fluids. While the current study did not successfully result in infection with PEDV in the positive control room, the results confirm that pigs were infected with PRRSV and SVA when viral inoculum was provided through feed.

The study herein was designed to validate the principle of extended storage for the inactivation of swine viruses, which has been repeated with multiple individual ingredients but has not been fully characterized for complete feed post-feed manufacture. A limitation of the current study is a limited sample size to fully characterize the effects of extended feed storage on nutritional, microbiological, and toxicological outcomes. Based on facility limitations and storage logistics, we were only able to include a limited amount of replication for evaluation of infectivity characteristics and microbiological/toxicological profiles. Follow-up research should be conducted to expand the base of knowledge of the effect of this practice on these key measurements of feed safety and quality. Based on the current data, there does not appear to be any significant negative consequences of 58 d storage at controlled anaerobic environmental conditions. 

This represents a significant advancement in the body of knowledge, as nearly all previous research with viral contaminants in swine feed have focused on ingredient storage. This experiment is the first to describe how real-world scale storage of complete swine feed can inactivate viral pathogens. While extended storage of complete feed is not practical in all situations, there is opportunity for its implementation in situations wherein a high degree of risk aversion is needed, such as biomedical research and/or high genetic value animals. Further research should be conducted to explore the stability of other pathogens which may be present in feed such as bacterial contamination or additional viral pathogens beyond those evaluated herein. Our study evaluated extended storage in anaerobic conditions established through the sublimation of dry ice and subsequent displacement of air from the storage containers and intermodal shipping containers. Additional research should be conducted to characterize the efficacy of this procedure compared to extended storage in non-anaerobic conditions as well as whether other compounds such as nitrogen could be used to create these anaerobic conditions. Furthermore, while we have established that a 58 d storage period is effective under the conditions evaluated, further research should be conducted to establish the optimum storage time based on pathogen inactivation and nutrient stability and feed quality characteristics. Nonetheless, the results provide the first evidence characterizing the potential applicability of this technique for the mitigation of viral contamination in swine complete feed.

## 5. Conclusions

In summary, storing feed for 58 d in controlled anaerobic environmental conditions of 23.9 °C did not result in microbiological or toxicological concerns and only resulted in minimal detrimental effects on nutrient stability. No evidence of infection with PEDV, PRRSV, or SVA was detected in the swine bioassay rooms fed inoculated feed following this 58 d storage period at 23.9 °C with the exception of the aerosol transmission of PRRSV via a documented biocontainment breach. Furthermore, if using extended storage for complete feed, it is recommended for this to be performed at controlled temperatures to maximize the consistency in viral inactivation. Ambient storage may be effective under certain conditions, but inactivation would be expected to be greatly reduced if the feed is stored at temperatures near or below freezing. Additional research should focus on evaluating the time necessary to store complete swine feed for inactivation of swine viruses with consideration given to shorter storage durations. Together, these results validate that the extended storage of complete swine feed can be a method to reduce risk associated with pathogen transmission through feed while having minimal effects on measures of nutritional quality. While this practice may not be applicable in commercial swine production, it may be considered as a means of improving the safety of swine feed for applications such as biomedical research and/or feeding of high genetic value animals wherein a high degree of risk aversion is required to maintain animal health.

## Figures and Tables

**Table 1:** Diet composition (as-fed basis) ^1.^

Item:	Swine Finishing Diet
Ingredient, %	
Corn	84.81
Soybean meal, 46.5% CP	7.35
Corn DDGS	5.00
Calcium carbonate	1.00
Salt	0.50
L-Lys HCl	0.37
Monocalcium phosphate, 21% P	0.25
Threonine ^1^	0.13
Vitamin premix ^2^	0.13
Trace mineral premix ^3^	0.13
L-Trp	0.02
Phytase ^4^	0.02
DL-Met	0.01
Dietary acidifier ^5^	0.30
Total	100
Calculated analysis, %	
CP	12.00
Ether extract	3.42
Ca	0.48
Available P	0.24

^1^ Thr-PRO (CJ, Downers Grove, IL, USA). ^2^ Each kilogram contains 1,653,468 IU vitamin A, 661,387 IU vitamin D3, 17,637 IU vitamin E, 1,323 mg menadione, 3,307 mg riboflavin, 11,023 mg pantothenic acid, 19,842 mg niacin, 13.2 mg vitamin B12. ^3^ Each kilogram contains 22 g Mn, 73 g Fe, 73 g Zn, 11 g Cu, 198 mg I, and 198 mg Se. ^4^ HiPhos 2700, DSM Nutritional Products, Parsippany, NJ. ^5^ Commercial blend of formic and propionic acid (pHorce, Anpario, Nottinghamshire, United Kingdom). Feed additive was included in virus-inoculated feed totes and feed totes used to assess microbiological, toxicological, and nutritional profiles. Remainder of feed did not contain feed additives and corn was added at the expense of the dietary acidifier.

**Table 2:** Effect of complete feed storage on detection of mycotoxins and microbial profile.

		D 0 ^1^		d 58	
Item	Limit of Detection ^2^	Sample 1	Sample 2	Controlled Storage	Ambient Storage
Mycotoxin analysis					
Aflatoxin B1	20	<20	<20	<20	<20
Aflatoxin B2	20	<20	<20	<20	<20
Aflatoxin G1	20	<20	<20	<20	<20
Aflatoxin G2	20	<200	<200	<200	<200
Fumonisin B1	200	<200	<200	<200	<200
Fumonisin B2	200	<200	<200	<200	<200
Fumonisin B3	200	<200	<200	<200	<200
HT-2	200	<20	<20	<20	<20
T-2	20	<200	<200	<200	<200
DAS	200	<20	<20	<20	<20
Ochratoxin A	20	<20	<20	<20	<20
Sterigmatocystin	20	<100	<100	<100	<100
Zearalenone	100	<200	<200	<200	<200
DON	200	<200	<200	<200	<200
15-ADON	200	<200	<200	<200	<200
3-ADON	200	<20	<20	<20	<20
Microbiology analysis					
Yeast, cfu/g	10 cfu/g	120	<10	<10	490
Mold, cfu/g	10 cfu/g	2800	2700	90	320
*Salmonella*, cfu/25 g	ND per 25 g	ND	ND	ND	ND
*E. coli* 0157:H7, cfu/25 g	ND per 25 g	ND	ND	ND	ND
*Staphylococcus aureus*, cfu/g	10 cfu/g	<10	<10	<10	<10
Aerobic plate count, cfu/g	10 cfu/g	5300	2000	260	2200
*Clostridium perfringens*, cfu/g	10 cfu/g	<10	<10	<10	<10

^1^ Two samples analyzed on d 0 representing two composite samples of the manufactured feed. Samples for d 58 controlled storage and d 58 ambient storage are the result of 1 sample collected from each storage condition. ^2^ For mycotoxin testing, limit of detection if practical quantification limit (PQL, µg/kg) as reported by North Dakota State University Veterinary Diagnostic Laboratory (Fargo, ND, USA). For microbiology testing, limit of detection as reported by Eurofins Microbiology Laboratories (Garden Grove, CA, USA).

**Table 3:** Effect of complete feed storage on quantification of dietary vitamins and phytase ^1^.

	d 0	d 58		
Item	Baseline	4 °C	Controlled Storage	Ambient Storage
*n*=	6	6	3	3
Phytase				
Claim, FYT/kg	500	500	500	500
Analyzed, FYT/kg	877 ± 113.5 ^a^	578 ± 54.7 ^b^	584 ± 85.2 ^b^	497 ± 43.8 ^b^
Percent of claim	175 ± 22.7 ^a^	116 ± 10.9 ^b^	117 ± 17.0 ^b^	99 ± 8.8 ^b^
Vitamin A				
Claim, IU/kg	4135	4135	4135	4135
Analyzed, IU/kg	2425 ± 634.0 ^c^	2702 ± 788.6 ^bc^	4174 ± 984.5 ^ab^	4571 ± 1015.4 ^a^
Percent of claim	59 ± 15.3 ^c^	65 ± 19.1 ^bc^	101 ± 23.8 ^ab^	111 ± 24.6 ^a^
Vitamin B5				
Claim, mg/kg	28	28	28	28
Analyzed, mg/kg	91.5 ± 10.7 ^a^	24.8 ± 12.6 ^b^	27.3 ± 4.2 ^b^	25.0 ± 2.6 ^b^
Percent of claim	327 ± 38.2 ^a^	89 ± 44.9 ^b^	98 ± 14.9 ^b^	89 ± 9.4 ^b^
Vitamin D3				
Claim, IU/kg	1654	1654	1654	1654
Analyzed, IU/kg	1459 ± 411.2	1539 ± 222.5	1587 ± 119.2	1659 ± 180.4
Percent of claim	88 ± 24.9	93 ± 13.5	96 ± 7.2	100 ± 10.9
Vitamin E				
Claim, IU/kg	44	44	44	44
Analyzed, IU/kg	48.8 ± 5.7	50.5 ± 2.1	45.0 ± 0.0	43.7 ± 2.5
Percent of claim	111 ± 13.0	115 ± 4.7	102 ± 0.0	99 ± 5.7

^1^ Samples analyzed by DSM Nutritional Products Technical Marketing Analytical Services Laboratory (Belvidere, NJ, USA). Differences between sample storage conditions for analyzed phytase or vitamin concentration and percentage of recovery relative to calculated claim was evaluated using a linear model. If results of the one-way ANOVA were *p* < 0.05, pairwise comparisons between storage conditions were performed and means lacking common superscript within analysis row differ (*p* < 0.05). Mean ± standard deviation.

**Table 4:** Effect of complete feed storage on serum antibody testing ^1^.

	0 dpi			27 dpi		
Item	PEDV	PRRSV	SVA	PEDV	PRRSV	SVA
Test room 1	0/10	0/10	0/10	0/10	0/10	0/10
Test room 2	0/9	0/9	0/9	0/9	0/9	0/9
Test room 3	0/10	0/10	0/10	0/10	0/10	0/10
Test room 4	0/9	0/9	0/9	0/9	0/9	0/9
Test room 5	0/11	0/11	0/11	0/11	10/11	0/11
Positive control	0/10	0/10	0/10	0/10	10/10	0/10

^1^ Samples analyzed by Iowa State Veterinary Diagnostic Laboratory (Ames, IA, USA). PEDV = porcine epidemic diarrhea virus, PRRSV = porcine reproductive and respiratory syndrome virus, SVA = senecavirus A, dpi = day post-inoculation.

## Data Availability

The data presented in this study are available on request from the corresponding author. The data are not publicly available due to [All data generated or analyzed during this study are included in this published article].
